# Editorial: Streptococci in infectious diseases – pathogenic mechanisms and host immune responses

**DOI:** 10.3389/fmicb.2022.988671

**Published:** 2022-07-20

**Authors:** Simone Bergmann, Marcus Fulde, Nikolai Siemens

**Affiliations:** ^1^Institute of Microbiology, Technische Universität Braunschweig, Braunschweig, Germany; ^2^Centre for Infection Medicine, Institute of Microbiology and Epizootics, Free University of Berlin, Berlin, Germany; ^3^Department of Molecular Genetics and Infection Biology, University of Greifswald, Greifswald, Germany

**Keywords:** streptococci, pathogenesis, immune evasion, infection, diseases

The genus *Streptococcus* encompasses over fifty species which are classified into alphabetical groups based on cell surface antigens according to Lancefield (1928). Although the majority of them are commensal part of the human or animal microbiota, they also cause diseases (Krzysciak et al., 2013). Most streptococcal infections are of a mild nature. However, these bacterial species also cause highly invasive diseases. These include but are not limited to necrotizing skin and soft tissue infections (NSTIs; *S. pyogenes*) (Siemens et al., 2020), pneumonia, sepsis, and meningitis (*S. pneumoniae, S. suis*) (Votsch et al., 2018; Steinert et al., 2020; Palmer and Kimmey, 2022), neonatal sepsis (*S. agalactiae*) (Armistead et al., 2019), and endocarditis (*S. anginosus*) (Reissmann et al., 2010). The invasiveness is linked to a plethora of bacterial as well as host factors. Virulence factors help pathogens to escape the host immune response while uncontrolled and excessive activation of host factors can aggravate the disease progression (Doran et al., 2016; Siemens et al., 2020; Steinert et al., 2020). Consequently, all these actions can result in substantial host tissue damage, bacterial dissemination, and subsequent death of the host.

For this Research Topic we collected eight papers, including seven original research and one review articles. The review and two additional research papers describe epidemiology and virulence determinants of *S. agalactiae* (group B streptococcus [GBS]). Furuta et al. discuss the clinical impact of neonatal GBS invasive infections. The authors highlight key aspects of GBS maternal-infant transmission, pathogen acquisition, GBS pathogenesis, and suggest that it will be a key for future therapies to identify crucial pathogenic mechanisms of GBS in infants. GBS produce membrane vesicles (MVs), which are implicated in disease pathogenesis (Armistead et al., 2021). McCutcheon et al. quantified MV production by different GBS isolates and examined protein composition. The study revealed that MV production and composition is strain dependent. They contain virulence and immunomodulatory factors. The authors conclude that GBS MVs potentially have lineage specific functions in virulence. The study by Jones et al. presents important epidemiological results. One hundred GBS isolates, 50 obtained from rectovaginal screening swabs of pregnant women and 50 from blood cultures of invasive infections, were characterized. Capsular genotype Ia was predominant in colonizing strains while genotype V was predominant in invasive strains. All isolates were susceptible to penicillin. Of concern, two isolates showed reduced susceptibility to ceftriaxone and were found to have unique alleles at *pbp2X* and *pbp1A*. Several studies identified point mutations within *pbp2x*, which were associated with reduced or non-susceptibility to β-lactam antibiotics in streptococci (Southon et al., 2020; Mcgee et al., 2021; Beres et al., 2022). Therefore, emergence of such clones warrants ongoing monitoring.

Since *S. pyogenes* causes acute infections in humans, it was not considered a major biofilm forming species. A recent study identified biofilms in 32% of NSTI patients, which might be of concern for treatment of these infections (Siemens et al., 2016). Therefore, Skutlaberg et al. analyzed biofilm forming capacity of 57 *S. pyogenes* NSTI isolates of different *emm*-types and related them to patient demographics and clinical variables. The study shows that *emm*1 strains possess the best biofilm forming capacity compared to other invasive *emm* types. However, the impact of biofilm formation on clinical outcomes remains uncertain and requires further studies.

Two research studies deal with cell wall associated components of *S. suis*. *S. suis* is a common swine pathogen. To escape immuno-clearance, streptococci modify LTA by incorporating D-alanine (Percy and Grundling, 2014). The study by Öhlmann et al. demonstrates that D-alanylation of LTAs is associated with reduced complement factor C3 deposition on *S. suis* surface, increased resistance to antimicrobial peptides, and reduced phagocytosis. The authors conclude that *S. suis* modifies its surface to avoid recognition and ingestion by phagocytes. The study by Cabezas et al. presents novel findings on substrate specificity of the cell wall anchored protein SntA. SntA is a phosphohydrolase, which hydrolyses c-di-AMP. In most bacteria, c-di-AMP is mainly involved in maintaining proper turgor pressure (Commichau and Stulke, 2018). However, when pathogens are intracellularly located, secreted c-di-AMP is detected by the host cells. This results in STING-dependent IFN induction by the host, which facilitates immuno-clearance. Therefore, it is of crucial importance for *S. suis* to express a high affinity hydrolase, which controls the amount of secreted c-di-AMP.

Bacteriocins are antimicrobial peptides produced by bacteria that target other, often closely related species, to gain an advantage within bacterial communities (Vogel and Spellerberg, 2021). *Streptococcus anginosus* produces angicin. Angicin inhibits other streptococcal species, Listeria spp., and various enterococci. The production of angicin is regulated by the quorum sensing system Sil which, among others, contains the genetic information for the CAAX protease SilX. It is known that streptococcal CAAX proteases from bacteriocin clusters are involved in immunity to bacteriocins, process them or act as receptors. Vogel et al. have found evidence for a new function of CAAX proteases: by processing SilCR, the signal peptide of the Sil system, SilX could now be less indicative of a possible auto-immunity or a direct bacteriocin interaction, but rather the basis for an indirect regulatory mechanism of bacteriocin production and processing.

Bloodstream infections by streptococci and/or pneumococci are often accompanied by damaged endothelium (Steinert et al., 2020). Therefore, it is of crucial importance to establish experimental *in vitro* models that mimic flow conditions. The paper by Kopenhagen et al. presents an elegant approach to study endothelial cell migration and proliferation as two major prerequisites for tissue regeneration under shear stress. This technique provides a powerful tool to analyze the impact of pneumococcal infections in real time and should be considered for future studies of any type of bloodstream infections.

In conclusion, the eight articles provide new exciting insights into the kaleidoscope of streptococcal pathogenesis and offer fundamental news with regard to the various scientific topics. We sincerely thank all authors and reviewers for their contributions to this Research Topic.

## Author contributions

All authors listed have made a substantial, direct, and intellectual contribution to the work and approved it for publication.

## Conflict of interest

The authors declare that the research was conducted in the absence of any commercial or financial relationships that could be construed as a potential conflict of interest.

## Publisher's note

All claims expressed in this article are solely those of the authors and do not necessarily represent those of their affiliated organizations, or those of the publisher, the editors and the reviewers. Any product that may be evaluated in this article, or claim that may be made by its manufacturer, is not guaranteed or endorsed by the publisher.
